# How Does Nerve Mechanical Interface Treatment Impact Pre-Surgical Carpal Tunnel Syndrome Patients? A Randomized Controlled Trial

**DOI:** 10.3390/jpm14080801

**Published:** 2024-07-29

**Authors:** Mar Hernández-Secorún, Hugo Abenia-Benedí, María Orosia Lucha-López, María Durán-Serrano, Javier Sami Hamam-Alcober, John Krauss, César Hidalgo-García

**Affiliations:** 1 Unidad de Investigación en Fisioterapia, Faculty of Health Science, Universidad de Zaragoza, 50009 Zaragoza, Spain; hugoabenia1@gmail.com (H.A.-B.); hidalgo@unizar.es (C.H.-G.); 2 Unit of Reconstructive Surgery of the Locomotor System, Hand-Microsurgery, Department of Orthopaedic Surgery and Traumatology, Hospital Universitario Miguel Servet, 50009 Zaragoza, Spain; mdurans@salud.aragon.es (M.D.-S.); jshamam@salud.aragon.es (J.S.H.-A.); 3 School of Health Sciences, Oakland University, Rochester, MI 48363, USA; krauss@oakland.edu

**Keywords:** carpal tunnel syndrome, conservative treatment, physical therapy, waiting lists

## Abstract

Background: Carpal tunnel syndrome (CTS) presents a high burden on the healthcare system. However, no alternative treatments are provided during the waiting period. In addition, the incidence of severe patients with comorbidities is underestimated. The aim of this study was to determine whether nerve mechanical interface treatment improves the symptoms, function, and quality of life in pre-surgical CTS patients. Methods: A randomized controlled trial and intention-to-treat analysis were carried out. Forty-two patients with an electrodiagnosis of carpal tunnel syndrome, included on the surgery waiting list of a public healthcare system, were analyzed. The intervention group (*n* = 20) received a 45 min session/per week of instrument-assisted manual therapy (diacutaneous fibrolysis) for 3 weeks. The Boston Carpal Tunnel Questionnaire (BCTQ) was the primary outcome. The symptoms, mechanical threshold, grip strength, mechanosensitivity of the median nerve, quality of life, and patient satisfaction were included as secondary outcomes. The control group (*n* = 22) remained on the waiting list. Results: The intervention seems to be beneficial for the BCTQ score (function and symptoms scale), pain, and mechanosensitivity after treatment, at the 3 and 6 months follow-up (*p* < 0.05). Kinesiophobia was improved at 6 months (*p* = 0.043; η^2^ = 0.10) and the mechanical threshold at the 3-month follow-up (*p* = 0.048; η^2^ = 0.10). No differences were identified for grip strength. At 6 months, the intervention group patients were satisfied (100%), as opposed to the controls, who felt that they had experienced a worsening of their condition (50.1%). Conclusions: Nerve mechanical interface treatment improved the symptoms, function, and quality of life in pre-surgical CTS patients. One hundred percent of the treated patients, characterized as moderate and severe CTS with associated comorbidities, were satisfied.

## 1. Introduction

Carpal tunnel syndrome (CTS) is the most prevalent focal mononeuropathy, constituting 90% of all neuropathy cases [1]. Carpal tunnel release (CTR) is one of the most frequently performed surgical procedures, with an estimated hospital discharge rate in Spain of 0.18–1000, for working patients [2]. CTR is an expensive procedure, contributing to the growth in hand surgery incidence at one of the fastest rates [3]. According to the Spanish public health service, the typical delay before CTR surgery, from 2019 to 2023, was over 5 months [4]. Individuals on the waiting list do not receive physiotherapy treatment during this timeframe. 

In recent years, some studies have looked at the impact of conservative treatment as an alternative to surgery. Fernández de las Peñas et al. [5] observed that a physiotherapy approach obtained similar effects as the surgical procedure after four years of follow-up. Moreover, it resulted in lower direct and indirect healthcare costs [6]. In another study, Lewis et al. [7] estimated that a program of education, splinting, and home exercise reduced the likelihood of surgery by 21% at 24 weeks. 

Nerve mechanical interface treatment is defined as the treatment of tissues surrounding the nerve, reducing nerve loading and improving their mobility [8]. Within this group, diacutaneous fibrolysis (DF) is an instrument-assisted manual therapy. It consists of the application of specially designed, hook-shaped steel instruments into the soft tissue surrounding the nerves, mobilizing the soft tissue with the brief traction of the instruments [9,10]. A recent meta-analysis has looked at the effectiveness of nerve mechanical interface treatment [11], showing that mechanical interface techniques are effective in improving pain and function in people with CTS. Jiménez del Barrio et al. [12,13] applied a protocol of manual and instrument-assisted (diacutaneous fibrolysis) soft tissue mobilization of the myofascias in the ventral forearm, ventral tendons, and fascia of the hand in patients with mild to moderate carpal tunnel syndrome compared to a control group. The protocol was applied during five sessions, which reduced the intensity of nocturnal pain and improved upper extremity functionality, the Boston Carpal Tunnel Questionnaire score, nerve conduction, and mechanosensitivity compared to the control group after the treatment [12]. However, patients with severe CTS and those with comorbidities have generally been excluded from participation in studies adopting a conservative approach to CTS [14]. CTS patients on the surgical waiting list often have certain characteristics, which are underrepresented in other studies [15]. Therefore, evidence of the potential effects of nerve mechanical interface treatment on patients with CTS that are on the surgical waiting list, is lacking.

This research aimed to determine whether nerve mechanical interface treatment improves the symptoms, function, and quality of life in patients with CTS that are waiting for surgery. In addition, this study will explore what patient characteristics are associated with the best outcomes from this treatment combination. 

## 2. Materials and Methods

### 2.1. Design

A randomized controlled trial was conducted in patients suffering from CTS, who were on the surgery waiting list of a Spanish public healthcare system. A medical team member checked the patient’s suitability for participant selection, and they were referred to the Faculty of Health Science. The selection criteria were revised, and a baseline assessment was performed by a researcher. Eligible participants were randomly allocated to the intervention or control group after the baseline assessment. The intervention consisted of an approach that included education, nerve mechanical interface treatment (diacutaneous fibrolysis), and self-mobilization of the ventral forearm, wrist, and hand myofascias. The control group received standard care for CTS. The randomization process was computer generated using Random.org, stratified according to the classification of the CTS severity (mild, moderate, and severe) [16], and concealed in sealed envelopes. After treatment, 3- and 6-month follow-ups were performed by an evaluator. This study was registered at www.clinicaltrials.gov, with the number NCT05130931, and approved by the Local Ethics Committee (CEICA), number 05/2021. A CONSORT statement was followed (Supplementary Materials, Table S1).

### 2.2. Participants and Centers

CTS patients on the surgical waiting list of a Spanish public healthcare system were recruited from May 2021 to November 2022. The patients came from Miguel Servet University Hospital and “San José” and “Ramón y Cajal” specialty medical centers. The inclusion criteria were: (1) patients >18 years old; (2) patients with nerve conduction studies that confirmed CTS, according to the Bland et al. classification [16]; (3) patients with more than three months of persistent CTS symptoms (paraesthesia, nocturnal symptoms…); (4) patients able to understand and perform all the assessment task; and (5) patients having signed the informed consent form.

The exclusion criteria included: (1) previous carpal tunnel release surgery on the homolateral upper limb; (2) previous history of traumatic injury of the homolateral upper limb; (3) diagnosis of other musculoskeletal or neurological pathologies that may contribute to the development of CTS; (4) steroid or physiotherapeutic treatment in the last six months; and (5) being pregnant.

### 2.3. Intervention

During the study, participants in both groups remained on the surgical waiting list. The project did not interfere with the surgical unit and the waiting list process, so patients in both groups could be called for surgery. 

#### 2.3.1. Intervention Group (IG)

One 45 min session per week for three weeks was administered individually at the Faculty of Health Science. Each one of the three sessions was composed of 15 min of education, 20 min of instrument-assisted manual therapy (diacutaneous fibrolysis) of the forearm, wrist, and hand soft tissues, and 10 min of instruction and supervision on self-mobilization for home-based self-mobilization. Home-based self-mobilization was prescribed to be performed 3 to 5 times a day. A physiotherapist with more than three years of clinical experience performed the intervention. One month of training was performed to standardize the intervention protocol. A detailed description of the intervention is available in the Supplementary Materials (Figure S1).

#### 2.3.2. Control Group (CG)

Patients in the CG received standard care, including remaining on the waiting list and following the recommendations prescribed by their surgeon. After the follow-up, CG participants were offered the protocol treatment given to the IG. 

### 2.4. Outcome Measures

Sociodemographic information was collected and is included in the Supplementary Materials (Table S2). 

#### 2.4.1. Primary Outcome

The Boston Carpal Tunnel Questionnaire (BCTQ) was used to assess the severity of the symptoms and current function. It is divided into two sub-scales. The symptom severity scale (SSS) scored from 0 to 55 and the function severity scale (FSS) scored from 0 to 45. Higher scores indicate greater symptom severity and lower function. The Spanish version was used. It is considered a valid and reliable instrument for CTS patients [17]. The questionnaire was self-administered through Google Forms.

#### 2.4.2. Secondary Outcome

The pain intensity, grip strength, mechanical sensory threshold, mechanosensitivity of the median nerve, kinesiophobia, and patient satisfaction, were assessed. 

The pain intensity was evaluated with a specific visual analogue scale (VAS). Subjects were shown a line with a length of 100 mm, with 0 being “no pain” and 100 being “maximum symptom experienced”. They were instructed to mark the average pain intensity they experienced during the last week [18]. 

Semmes–Weinstein monofilaments (Touchtest, Monterey, CA, USA) were used to assess the mechanical threshold of the hand. Testing was performed at the midpoint of the distal phalanx of the five fingers. The assessment began with the 2.83 (0.07 g) filament, which is considered normal. The tool consists of 16 monofilaments: four representing normal sensitivity (1.65 to 2.85) and twelve representing hyposensitivity (3.22 to 6.65). During the test, the patient laid in the supine position with their eyes closed, arms by their sides, and palms facing upward. Each finger received five stimuli and at least two had to be felt. The sequence of the finger stimulation was randomized to prevent the patient learning the order. Once the patient identified two stimuli, the corresponding filament number was recorded [19,20]. The final result was the average of the mechanical thresholds, indicated by the filament number, across all five fingers.

Hand grip strength was measured in kilograms, using a hand dynamometer (Jamar, Patterson Medical, Chicago, IL, USA). Patients were positioned with their elbow supported at 90° of flexion and in a neutral pronation–supination position. They were instructed to squeeze the dynamometer as hard as possible, within their pain tolerance, for seven seconds, in three separate trials. The average of the maximum strength recorded from the three trials was taken as the final value [21].

The Upper Limb Neurodynamic Test 1 (ULNT1) was used to evaluate the mechanosensitivity of the median nerve. Subjects were laid in the supine position, with straight legs, and at rest. The procedure followed the method described by Shacklock et al. [22]: (1) 90° shoulder abduction; (2) shoulder external rotation; (3) forearm supination; (4) wrist and finger extension; and (5) elbow extension. When symptoms appeared, the movement was halted, and structural differentiation was conducted by moving the region furthest from the symptomatic area. Elbow extension was measured with a conventional goniometer (Jamar, Sammons Court, Bolingbrook, IL, USA), from 90° flexion to maximal extension. A greater number of degrees indicated a higher tolerance of the median nerve to mechanical stimuli [23,24,25].

Kinesiophobia was evaluated using the Tampa Scale short form, which consists of 11 questions, each rated from 1 to 4. Scores range from 11 to 44 points, with higher scores indicating greater levels of kinesiophobia. The scale has been shown to have good psychometric properties [26,27].

Finally, patient satisfaction was assessed by the Patient Global Impression of Improvement (PGI-I) scale. This scale consists of a single question that asks the patient to rate the relief obtained from the following treatment using a seven-point Likert scale: (1) very much improved; (2) much improved; (3) minimally improved; (4) no change; (5) minimally worse; (6) much worse; or (7) very much worse [28]. 

All the outcomes were assessed at the baseline, after treatment, and at 3-month and 6-month follow-ups, except the PGI-I scale (at 3 and 6 months). 

### 2.5. Data Analysis

#### 2.5.1. Sample Size Calculation

The sample size was calculated using the G*Power v3.1 program, using the BCTQ score as the primary outcome. The effect size (ES) was determined using the outcomes described by Jiménez del Barrio et al. [29], namely the ES = 0.76, and the correlation between the repeated measurements was assumed to be 0.5. The number of measurements used in the calculation was 3 (post-intervention, 3 months, and 6 months) for the two groups. With a statistical power of 0.80 and an alpha level of 0.05, a total sample size of 18 patients was estimated. To address any potential loss of participants during the follow-up period, an additional 30% was added to the recruitment, leading to a total sample of 24 participants (12 per group).

#### 2.5.2. Statistical Analysis

Statistical analysis was performed using the Statistics Package for Social Science (SPSS v26, IBM Inc., Armonk, NY, USA). Histograms and the Shapiro–Wilk test were used to assess the data normality. Normally distributed variables were presented as the mean and standard deviation. The median and interquartile range were used to describe the quantitative variables with a non-normal distribution. For qualitative variables, the absolute frequencies and percentages were calculated. The statistical analysis was performed on an intention-to-treat basis (Little’s missing completely at random test and expectation maximization).

A general linear model of repetitive measures was performed for between-group changes for the post-treatment (T1), 3-month (T2), and 6-month (T3) follow-up, in regard to the primary and secondary outcomes. The model was adjusted for the individual’s baseline value by including it as a covariate during the general linear model analysis (ANCOVA). If the assumption of sphericity was not met, the Greenhouse–Geisser correction was applied for interpretation. Upon the identification of a statistically significant effect, a post hoc analysis was conducted with the Bonferroni correction, to account for multiple comparisons. Next, a second general linear model of repetitive measures was performed that also considered the sociodemographic variables as between-subject factors or covariates. A post hoc analysis was performed when significant statistical value was found between the dependent variable and the sociodemographic variable. For the patient satisfaction, Fisher’s exact test was performed. The effect sizes were calculated using eta squared (η^2^). An effect size > 0.14 was considered large; around 0.06, intermediate; and <0.01, small [30]. The significance level was set at *p* > 0.05.

## 3. Results

**Figure 1:**
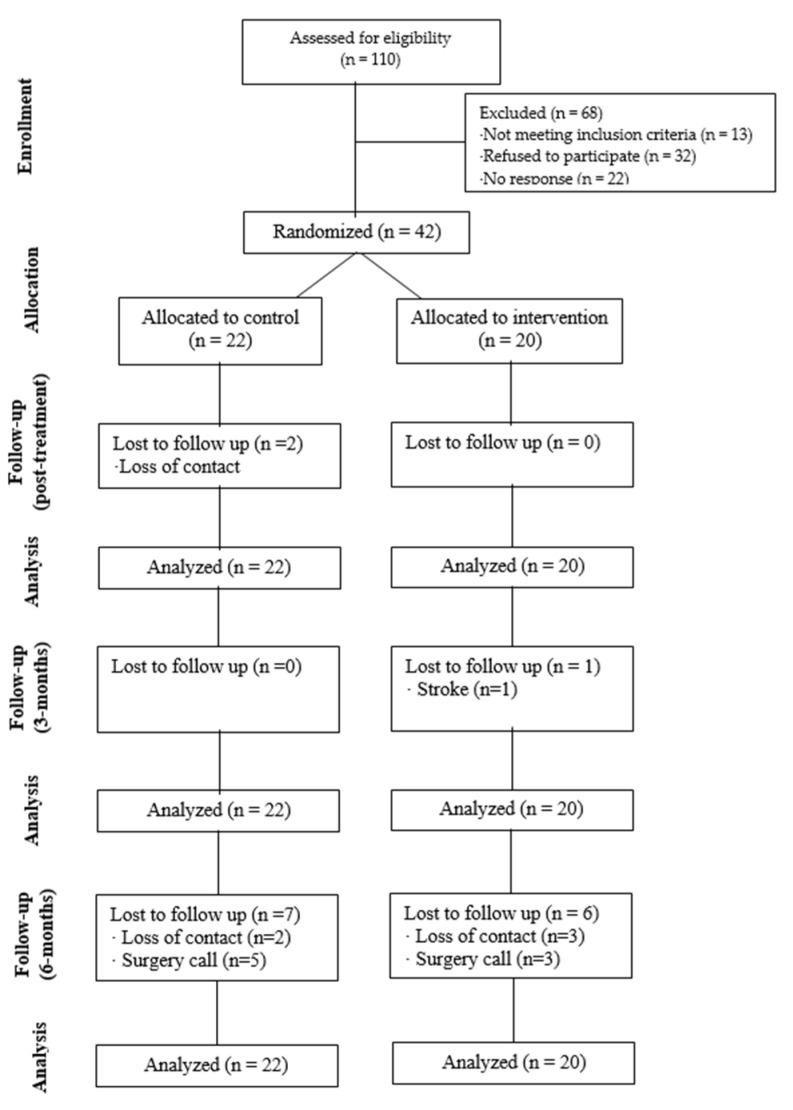
CONSORT diagram.

Between May 2021 and November 2022, 110 patients from Miguel Servet University Hospital, Ramón y Cajal, and San José specialized medical centers were assessed for eligibility. After assessing the patients in terms of the selection criteria, 68 patients were excluded for not meeting the inclusion criteria or refusing to participate. Finally, 42 patients were found to be suitable for randomization into the CG (*n* = 22) or IG (*n* = 20). During the follow-up, nine participants in the CG and seven participants in the IG did not complete the process, representing 38% of the sample. The CONSORT diagram is shown in Figure 1.

There were no significant differences between the groups except for the BMI (*p* = 0.036). All other sociodemographic characteristics were balanced (Table 1). The baseline primary and secondary outcomes are shown in the Supplementary Materials (Table S3). 

We analyzed the data collected at the baseline in a published correlational study [15]. Considering the large number of outcome measures, the different study design, and the main study purposes, the correlation analysis was examined in a previous publication.

### 3.1. Between-Group Analyses

#### 3.1.1. Boston Carpal Tunnel Questionnaire

**Figure 2:**
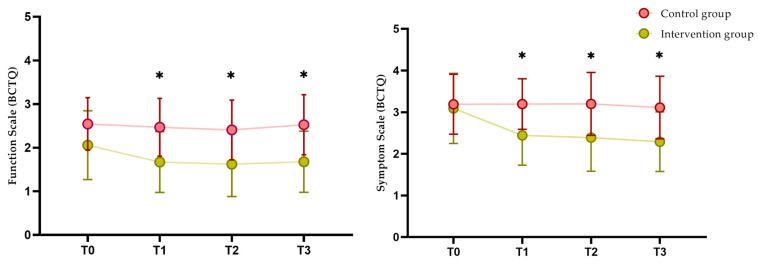
Boston Carpal Tunnel Questionnaire (BCTQ) between-group comparison. * Comparison between groups using repeated measures generalized linear model (*p* < 0.05).

The IG participants reported better results on the SSS and FSS of the BCTQ than the CG participants. Statistically significant differences were found between the groups after treatment (SSS: *p* < 0.001; η^2^ = 0.37/FSS: *p* = 0.003; η^2^ = 0.20), at the three-month (SSS: *p* = 0.001; η^2^ = 0.24/FSS: *p* = 0.01; η^2^ = 0.16) and six-month (SSS: *p* = 0.001; η^2^ = 0.25/FSS: 0.004; η^2^ = 0.23) follow-up (Figure 2).

#### 3.1.2. Secondary Outcomes

The IG also reported decreased pain intensity after treatment (*p* < 0.001; η^2^ = 0.51), at the three-month (*p* = 0.001; η^2^ = 0.25) and six-month follow-up (*p* < 0.001; η^2^ = 0.36) compared to the CG (Table 2). In addition, the hand mechanical threshold improved for the IG at three months (*p* = 0.048; η^2^ = 0.10), although the hand grip strength was not different between the groups. The mechanosensitivity of the median nerve also improved in the IG after treatment (*p* < 0.001; η^2^ = 0.70), at the three-month (*p* < 0.001; η^2^ = 0.47) and six-month (*p* < 0.001; η^2^ = 0.53) follow-up (Table 2). Finally, a reduction in kinesiophobia in the IG was found, compared to the CG, following treatment (*p* = 0.016; η^2^ = 0.14) and at the six-month follow-up (*p* = 0.043; η^2^ = 0.10) (Table 2). 

### 3.2. Influence of Sociodemographic Variables

**Figure 3:**
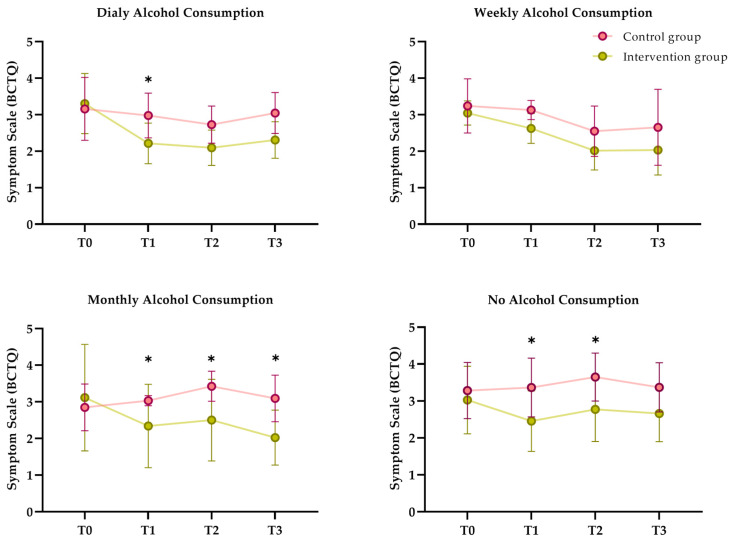
Symptom severity scale of the Boston Carpal Tunnel Questionnaire between-group comparison influenced by alcohol consumption. * Comparison between groups using repeated measures generalized linear model (*p* < 0.05).

Two sociodemographic variables (actual profession and alcohol consumption) influenced both the primary and secondary outcome variables (Figure 3 and Table S4).

The symptom severity scale was influenced by alcohol consumption. No improvement in symptoms was observed in the IG for those patients who consumed more alcohol, on a weekly or daily basis, except for just after treatment in those who consumed alcohol daily (*p* = 0.024; η^2^ = 0.15) (Figure 3).

Participants in the IG who did not consume alcohol or who consumed alcohol on a monthly basis demonstrated reduced symptoms compared to the CG for all follow-up periods. The patients that did not consume alcohol did not show any statistical differences between the groups at the six-month follow-up (*p* = 0.075; η^2^ = 0.09).

The working participants included in the IG showed significantly better results than the CG after treatment (*p* < 0.001; η^2^ = 0.50), at the three-month (*p* = 0.001; η^2^ = 0.26) and six-month (*p* < 0.001; η^2^ = 0.32) follow-up in terms of pain intensity. However, participants in the IG who were non-workers, were better with respect to the subjects in the CG, but only immediately after treatment (*p* = 0.001; d = 0.25). All participants were primarily employed in professions requiring physical labor (Table S4).

### 3.3. Patient Satisfaction

**Figure 4:**
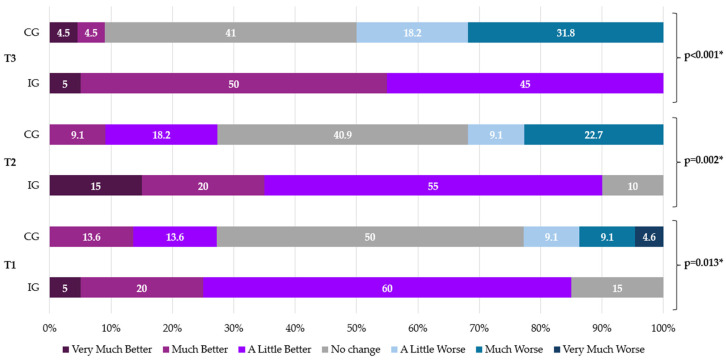
Patient satisfaction (PGI-I). CG: control group; IG: intervention group. * Fisher’s exact test.

After treatment, 72.5% of the CTS patients in the CG reported no improvement or a worsening PGI-I, compared to the IG, of which 15% reported no change and 85% reported an improvement (*p* = 0.013). At three months, 90% of the IG perceived an improvement, compared to the CG, in which 27.2% perceived an improvement (*p* = 0.002). At six months, 100% of the IG perceived an improvement after treatment, compared to 50% of the CG who reported worsening symptoms, and 40.9% who reported no change (*p* < 0.001). At all the follow-ups, a small percentage of patients in the CG showed some perception of an improvement (Figure 4).

## 4. Discussion

This randomized controlled trial showed that nerve mechanical interface treatment improved the symptoms, function, and quality of life of pre-surgical CTS patients immediately after, and at the three-month and six-month follow-ups, after the end of the intervention. Patients with CTS, placed on waiting lists, often experience long waiting times [31]. The longer a person experiences CTS symptoms, the more delayed and incomplete their recovery may be [32]. To our knowledge, this is the first study to analyze the effects of instrument-assisted manual therapy (fibrolysis diacutaneous) in moderate and severe CTS patients awaiting surgery.

An improvement in the BCTQ score was observed in the IG compared to the CG. This is consistent with prior studies [33,34,35], where the mobilization of the adjacent tissues to the median nerve improved the FSS and SSS of the BCTQ. While consistent with prior research, the improvements noted in our study are greater. This can be explained by the larger treatment area used in this study, where the tissues adjacent to the median nerve were mobilized in the forearm, hand, and wrist, whereas comparable studies performed treatment on the wrist only. It should be noted that all the comparable studies only included the immediate effects, except Günay et al. [33], who also included a three-month follow-up.

In addition to significant differences in favor of the intervention for the BCTQ, there were also significant differences in the pain intensity, mechanosensitivity, and kinesiophobia (except at the three-month follow-up). Jiménez del Barrio et al. [12,36] observed similar effects for diacutaneous fibrolysis treatment in mild and moderate CTS patients. Unique to this study is the use of a three-session treatment, for moderate and severe CTS, with measures taken immediately after the intervention, and at three-month and six-month follow-ups.

While no improvements in the grip strength and hand mechanical threshold were noted in this study, this is consistent with the studies by Mamipour et al. [37] and Ceylan et al. [38] who also did not observe improvements in strength. While the finding is not consistent with Fernández de las Peñas et al. [39], who observed that the thumb–index finger pinch strength improved one month after treatment in the physiotherapy approach group, compared to the surgical group, and the gain was similar up to the one-year follow-up. However, although grip strength is closely related to CTS, pinch strength allows better identification of CTS due to its innervation of the thenar muscles.

To our surprise, neither the severity nor the presence of systemic pathologies influenced the results of this study. However, work and alcohol consumption did. CTS is strongly related to the type and intensity of the work performed by an individual. Exposure to working conditions with high levels of repetition, velocity, and a combination of multiple physical exposures increased the risk of developing CTS [40]. This may explain why working participants improved more than non-working participants, since they were not exposed to the same type and duration of demanding activities as non-working patients, who were unable to work due to CTS. Participants in this study who drank alcohol frequently responded similarly, regardless of the group to which they were assigned. One explanation may be that alcohol modulates the synaptic plasticity of neurotransmitter systems, such as the glutamatergic, GABAergic, or endocannabinoid systems [41], which may interfere with the effect of the treatment.

Finally, the satisfaction of the patients who received the intervention was significantly higher than the satisfaction of those who received standard care, indicating that their condition worsened over time. Lewis et al. [7] also observed higher satisfaction in patients suffering from CTS, who were on the surgical waiting list, who received education, splinting, and who took part in home-based exercise. However, in Lewis et al.’s study, the satisfaction decreased at follow-up.

This study demonstrates that patients with moderate to severe CTS, with/or without comorbidities, benefit from conservative treatment based on education and manual and instrument-assisted (diacutaneous fibrolysis) soft tissue mobilization of the myofascia in the ventral forearm, ventral tendons, and fascia of the hand. However, this study has some limitations. There were many dropouts, especially from three to six months after treatment, since this was the time when patients were called for surgery. Some patients were called for surgery before the end of the study, and for fear of relapse and losing their place on the waiting list, they underwent surgery, regardless of any improvement. In addition, the small number of participants limits the ability of this study to develop a profile of patients who may benefit the most from this specific treatment protocol. Another limitation is that the mechanical threshold was assessed from the first to the fifth finger, which may have generated a bias in the measurement if the patient experienced an unknown ulnar compression. Developing clinical guidelines to identify individuals who would benefit from conservative or surgical intervention, as a first line of action in more severe patients with comorbidities, would be useful. Physiotherapy may be a viable alternative for treating entrapment neuropathy, reducing the number of patients and, subsequently, the time spent by surgical candidates on the waiting list [7,39]. This is especially important since the longer a patient experiences symptoms, the slower and less complete their recovery from CTS [42].

## 5. Conclusions

In summary, a protocol based on nerve mechanical interface treatment improved the symptoms, function, and quality of life of pre-surgical CTS patients. One hundred percent of the treated patients were satisfied and perceived improvements in their symptoms, unlike the CG, who perceived a worsening of their condition. It should be noted that the participants in this study were experiencing moderate to severe CTS with associated comorbidities, factors which would typically be exclusion criteria in non-surgical studies.

## Figures and Tables

**Table 1:** Baseline sociodemographic characteristics.

	Intervention Group (*n* = 20)	Control Group (*n* = 22)	*p*-Value
Sex; *n* (%)			0.721
*Male*	8 (40%)	10 (45.50%)	
*Female*	12 (60%)	12 (54.50%)	
Age (years)	57.62 ± 8.24	60.37 ± 15.80	0.479
BMI (Kg·m^2^)	31.16 ± 5.93	27.56 ± 4.84	0.036
Time with symptoms (years)	4.00 (2.60)	3.80 (3.00)	0.563
Severity; *n* (%)			0.349
*Mild*	0 (0%)	0 (0%)	
*Moderate*	5 (25%)	3 (13.60%)	
*Severe*	15 (75%)	19 (86.40%)	
Bilaterality of symptoms; *n* (%)			0.449
*Yes*	18 (90%)	18 (81.80%)	
*No*	2 (10%)	4 (18.20%)	
Patient’s perception of the etiology cause; *n* (%)			0.758
*None*	4 (20%)	5 (22.70%)	
*Work*	13 (65%)	12 (54.50%)	
*Age*	1 (5%)	1 (4.50%)	
*Comorbidities*	1 (5%)	2 (9.10%)	
*Other*	2 (10%)	2 (9.10%)	
Presence of comorbidities; *n* (%)			0.067
*Yes*	16 (80%)	17 (77.30%)	
*No*	4 (20%)	5 (22.70%)	
Number of comorbidities; *n* (%)			0.572
*None*	5 (25%)	5 (22.70%)	
*One*	8 (40%)	5 (22.70%)	
*Two or more*	7 (35%)	12 (54.60%)	
Physical activity; *n* (%)			0.303
*150–300 min/week moderate or 75–150 min/week of vigorous aerobic activity*	6 (30%)	10 (45.50%)	
*None*	14 (70%)	12 (54.50%)	
Actual profession; *n* (%)			0.366
*Active*	13 (65%)	11 (50%)	
*Inactive*	7 (35%)	11 (50%)	
Work status; *n* (%)			0.297
*Employed*	13 (65%)	11 (50%)	
*Off work*	1 (5%)	2 (9.10%)	
*Unemployed*	3 (15%)	1 (4.50%)	
*Retired*	3 (15%)	8 (36.40%)	
Alcohol consumption; *n* (%)			0.846
*Daily*	3 (15%)	4 (18.20%)	
*Weekly*	6 (30%)	5 (22.70%)	
*Monthly*	4 (20%)	3 (13.60%)	
*No*	7 (35%)	10 (45.50%)	
Tabacco consumption; *n* (%)			0.580
*Yes*	16 (80%)	19 (86.40%)	
*No*	4 (20%)	3 (13.60%)	

Data are shown as mean ± standard deviation or median (interquartile range).

**Table 2:** Between-group comparisons in primary and secondary outcomes.

		T1—After Treatment				T2—3 Months				T3—6 Months			
		Mean ± SD	F	*p*-Value	η^2^ Power	Mean ± SD	F	*p*-Value	η^2^ Power	Mean ± SD	F	*p*-Value	η^2^ Power
Boston Carpal Tunnel Questionnaire													
*Symptom Severity Scale (1–5)*	CG	3.20 ± 0.61	22.93	**<0.** 001 *	0.37 0.99	3.20 ± 0.76	11.99	0.001 *	0.24 0.92	3.11 ± 0.75	13.24	0.001 *	0.25 0.94
	IG	2.45 ± 0.72				2.39 ± 0.81				2.29 ± 0.71			
*Function Severity Scale (1–5)*	CG	2.47 ± 0.66	9.72	0.003 *	0.20 0.86	2.41 ± 0.68	7.44	0.010 *	0.16 0.76	2.53 ± 0.69	8.47	0.006 *	0.23 0.81
	IG	1.67 ± 0.70				1.62 ± 0.74				1.68 ± 0.70			
Pain intensity (0–100)	CG	48.2 ± 23.4	40.39	**<0.** 001 *	0.51 0.99	54.0 ± 24.2	12.63	0.001 *	0.25 0.93	56.4 ± 24.6	21.56	**<0.** 001 *	0.36 0.99
	IG	15.7 ± 11.6				28.4 ± 23.5				24.0 ± 20.2			
Hand mechanical threshold (1.65–6.65)	CG	3.29 ± 0.54	1.82	0.186	0.04 0.26	3.24 ± 0.49	4.17	0.048 *	0.10 0.51	3.10 ± 0.42	3.53	0.068	0.08 0.45
	IG	3.11 ± 0.80				2.96 ± 0.64				2.87 ± 0.46			
Hand grip strength (Kg)	CG	20.6 ± 10.0	0.054	0.817	0.01 0.06	21.1 ± 10.7	0.012	0.914	0.01 0.05	20.3 ± 9.5	3.72	0.061	0.09 0.47
	IG	24.9 ± 10.8				24.6 ± 9.3				26.7 ± 8.4			
ULNT1 (°)	CG	92.5 ± 7.9	91.61	**<0.** 001 *	0.70 0.99	91.8 ± 7.0	34.68	**<0.** 001 *	0.47 0.99	91.7 ± 3.6	44.12	**<0.** 001 *	0.53 0.99
	IG	140.4 ± 19.6				128.9 ± 24.6				125.6 ± 21.2			
Tampa Scale (11–44)	CG	28.5 ± 8.6	6.32	0.016 *	0.14 0.69	27.0 ± 9.1	1.36	0.250	0.25 0.21	27.4 ± 8.0	4.39	0.043 *	0.10 0.53
	IG	23.3 ± 6.2				23.3 ± 7.0				22.4 ± 25.2			

ULNT1: Upper limb tension test 1. Data are shown as mean ± standard deviation. * Comparison between groups using repeated measures generalized linear model (*p* < 0.05).

## Data Availability

The original contributions presented in the study are included in the article/Supplementary Materials, further inquiries can be directed to the corresponding author/s.

## Supplementary Materials

The following supporting information can be downloaded at: https://www.mdpi.com/article/10.3390/jpm14080801/s1, Table S1: CONSORT statement; Figure S1: Physiotherapy intervention; Table S2: Sociodemographic outcomes; Table S3: Baseline primary and secondary outcomes; Table S4: Between-group comparisons in primary and secondary outcomes, including significant sociodemographic characteristics.

## References

[1] Sevy J.O., Sina R.E., Varacallo M. (2023). Carpal Tunnel Syndrome.

[2] García-Gómez M., Urbanos-Garrido R., Castañeda-López R., López-Menduíña P., Losada V. (2012). Coste sanitario del asma, cáncer de vejiga, tunel carpiano y otra patología osteoarticular atribuible al trabajo en españa en 2008. Rev. Esp. Salud Publica.

[3] Bernstein D.N.M., Gruber J.S., Merchan N., Garcia J.B., Harper C.M., Rozental T.D. (2020). What Factors Are Associated with Increased Financial Burden and High Financial Worry for Patients Undergoing Common Hand Procedures?. Clin. Orthop. Relat. Res..

[4] Gobierno de Aragón Lista de Espera 2023. https://leweb.salud.aragon.es/listaespera/estadis.do.

[5] Fernández-De-Las-Peñas C., Arias-Buría J.L., A Cleland J., A Pareja J., Plaza-Manzano G., Ortega-Santiago R. (2020). Manual Therapy Versus Surgery for Carpal Tunnel Syndrome: 4-Year Follow-Up from a Randomized Controlled Trial. Phys. Ther..

[6] Fernández-De-Las-Peñas C., Ortega-Santiago R., Díaz H.F.-S., Salom-Moreno J., Cleland J.A., Pareja J.A., Arias-Buría J.L. (2019). Cost-effectiveness evaluation of manual physical therapy versus surgery for carpal tunnel syndrome: Evidence from a randomized clinical trial. J. Orthop. Sports Phys. Ther..

[7] Lewis K.J., Ross L., Coppieters M.W., Vicenzino B., Schmid A.B. (2016). Education, night splinting and exercise versus usual care on recovery and conversion to surgery for people awaiting carpal tunnel surgery: A protocol for a randomised controlled trial. BMJ Open.

[8] Butler D.S. (1989). Adverse Mechanical Tension in the Nervous System: A Model for Assessment and Treatment. Aust. J. Physiother..

[9] Veszely M., Guissard N., Duchateau J. (2000). Contribution à l ’ étude des effets de la fibrolyse diacutanée sur le triceps sural. Ann. Kinésithér.

[10] Lucha-López M.O., Hidalgo-García C., Monti-Ballano S., Márquez-Gonzalvo S., Krauss J., Tricás-Vidal H.J., Tricás-Moreno J.M. (2023). Diacutaneous Fibrolysis: An Update on Research into Musculoskeletal and Neural Clinical Entities. Biomedicines.

[11] Prat P.I., Milan N., Huber J., Ridehalgh C. (2024). The effectiveness of nerve mechanical interface treatment for entrapment neuropathies in the limbs: A systematic review with metanalysis. Musculoskelet. Sci. Pract..

[12] del Barrio S.J., de Miguel E.E., Gracia E.B., Garay M.H., Moreno J.M.T., García C.H. (2018). Effects of diacutaneous fibrolysis in patients with mild to moderate symptomatic carpal tunnel syndrome: A randomized controlled trial. Clin. Rehabil..

[13] Jiménez-Del-Barrio S., Ceballos-Laita L., Bueno-Gracia E., Rodríguez-Marco S., Caudevilla-Polo S., Estébanez-De-Miguel E. (2022). Diacutaneous Fibrolysis Intervention in Patients with Mild to Moderate Carpal Tunnel Syndrome May Avoid Severe Cases in Elderly: A Randomized Controlled Trial. Int. J. Environ. Res. Public Health.

[14] Hernández-Secorún M., Montaña-Cortés R., Hidalgo-García C., Rodríguez-Sanz J., Corral-De-Toro J., Monti-Ballano S., Hamam-Alcober S., Tricás-Moreno J.M., Lucha-López M.O. (2021). Effectiveness of conservative treatment according to severity and systemic disease in Carpal Tunnel Syndrome: A systematic review. Int. J. Environ. Res. Public Health.

[15] Hernández-Secorún M., Abenia-Benedí H., Lucha-López M.O., Durán-Serrano M., Hamam-Alcober J.S., Krauss J., Booth-Smith C., Hidalgo-García C. (2024). Understanding the Mechanosensitivity of the Median Nerve in Pre-Surgical Carpal Tunnel Syndrome Patients: A Correlational Study. Brain Sci..

[16] Bland M. (2000). Producing benchmarks for clinical practice. Prof. Nurse.

[17] Oteo-Álvaro Á., Marín M.T., Matas J.A., Vaquero J. (2016). Validación al castellano de la escala Boston Carpal Tunnel Questionnaire. Med. Clin..

[18] Jensen M.P., A Turner J., Romano J.M., Fisher L.D. (1999). Comparative reliability and validity of chronic pain intensity measures. Pain.

[19] Jerosch-Herold C., Shepstone L., Miller L., Chapman P. (2011). The responsiveness of sensibility and strength tests in patients undergoing carpal tunnel decompression. BMC Musculoskelet. Disord..

[20] MacDermid J.C., Doherty T. (2004). Clinical and electrodiagnostic testing of Carpal Tunnel Syndrome: A narrative review. J. Orthop. Sports Phys. Ther..

[21] Trampisch U.S., Franke J., Jedamzik N., Hinrichs T., Platen P. (2012). Optimal jamar dynamometer handle position to assess maximal isometric hand grip strength in epidemiological studies. J. Hand Surg. Am..

[22] Shacklock M. (2009). Response to Butler and Coppieters 2007, Letter to the Editor: Clinical neurodynamics—Throwing the baby out with the bath water. Man. Ther..

[23] Bueno-Gracia E., Tricás-Moreno J.M., Fanlo-Mazas P., Malo-Urriés M., Haddad-Garay M., Estébanez-De-Miguel E., Hidalgo-García C., Krauss J.R. (2016). Validity of the Upper Limb Neurodynamic Test 1 for the diagnosis of Carpal Tunnel Syndrome. The role of structural differentiation. Man. Ther..

[24] Carla V., Laura C., Andrew G., Filomena M., Sergio P., Carlotta V., Paolo P. (2010). The Upper Limb Neurodynamic Test 1: Intra- and Intertester Reliability and the Effect of Several Repetitions on Pain and Resistance. J. Manip. Physiol. Ther..

[25] Vanti C., Bonfiglioli R., Calabrese M., Marinelli F., Guccione A., Violante F.S., Pillastrini P. (2011). Upper Limb Neurodynamic Test 1 and symptoms reproduction in carpal tunnel syndrome. A validity study. Man. Ther..

[26] French D.J., France C.R., Vigneau F., French J.A., Evans T.R. (2007). Fear of movement/(re)injury in chronic pain: A psychometric assessment of the original English version of the Tampa scale for kinesiophobia (TSK). Pain.

[27] Eiger B., Errebo M., Straszek C.L., Vaegter H.B. (2022). Less is more: Reliability and measurement error for three versions of the Tampa Scale of Kinesiophobia (TSK-11, TSK-13, and TSK-17) in patients with high-impact chronic pain. Scand. J. Pain.

[28] Guy W. (1976). ECDEU Assessment Manual for Psychopharmacology: Revised.

[29] Jiménez-del-Barrio S. (2016). Efectos del tratamiento fisioterápico mediante Fibrolisis Diacutánea en pacientes con Síndrome del Túnel Carpiano. Ph.D. Thesis.

[30] Pierce C.A., Block R.A., Aguinis H. (2004). Cautionary Note on Reporting Eta-Squared Values from Multifactor ANOVA Designs. Educ. Psychol. Meas..

[31] Abásolo I., Barber P., López-Valcárcel B.G., Jiménez O. (2014). Real waiting times for surgery. Proposal for an improved system for their management. Gac. Sanit..

[32] Masud M., Rashid M., Malik S.A., Khan M.I., Sarwar S.-U. (2019). Does the Duration and Severity of Symptoms Have an Impact on Relief of Symptoms after Carpal Tunnel Release?. J. Brachial Plex. Peripher. Nerve Inj..

[33] Gunay B., Alp A. (2015). The effectiveness of carpal bone mobilization accompanied by night splinting in idiopathic carpal tunnel syndrome. Turk J. Phys. Med. Rehabil..

[34] Shem K., Wong J., Dirlikov B. (2020). Effective self-stretching of carpal ligament for the treatment of carpal tunnel syndrome: A double-blinded randomized controlled study. J. Hand Ther..

[35] Talebi G.A., Saadat P., Javadian Y., Taghipour M. (2020). Comparison of two manual therapy techniques in patients with carpal tunnel syndrome: A randomized clinical trial. Casp J. Intern. Med..

[36] del Barrio S.J., Ceballos-Laita L., Bueno-Gracia E., Rodríguez-Marco S., Haddad-Garay M., Estébanez-De-Miguel E. (2021). Effects of Diacutaneous Fibrolysis on Mechanosensitivity, Disability, and Nerve Conduction Studies in Mild to Moderate Carpal Tunnel Syndrome: Secondary Analysis of a Randomized Controlled Trial. Phys. Ther..

[37] Mamipour H., Negahban H., Aval S.B., Zaferanieh M., Moradi A., Kachooei A.R. (2023). Effectiveness of physiotherapy plus acupuncture compared with physiotherapy alone on pain, disability and grip strength in people with carpal tunnel syndrome: A randomized clinical trial. J. Bodyw. Mov. Ther..

[38] Ceylan İ., Büyükturan Ö., Aykanat Ö., Büyükturan B., Şaş S., Ceylan M.F. (2023). The effectiveness of mobilization with movement on patients with mild and moderate carpal tunnel syndrome: A single-blinded, randomized controlled study. J. Hand Ther..

[39] Fernández-De-Las-Peñas C., Cleland J., Palacios-Ceña M., Fuensalida-Novo S., Pareja J.A., Alonso-Blanco C. (2017). The Effectiveness of Manual Therapy Versus Surgery on Self-reported Function, Cervical Range of Motion, and Pinch Grip Force in Carpal Tunnel Syndrome: A Randomized Clinical Trial. J. Orthop. Sports Phys. Ther..

[40] Gerger H., Macri E.M., Jackson J.A., Elbers R.G., van Rijn R., Søgaard K., Burdorf A., Koes B., Chiarotto A. (2024). Physical and psychosocial work-related exposures and the incidence of carpal tunnel syndrome: A systematic review of prospective studies. Appl. Ergon..

[41] Marcos A., Ballesteros-Yáñez I., Castillo-Sarmiento C.A., Pardo F., Roura-Martínez D., Muñoz-Rodríguez J.R., Higuera-Matas A., Ambrosio E. (2022). The interactions of alcohol and cocaine regulate the expression of genes involved in the GABAergic, glutamatergic and endocannabinoid systems of male and female rats. Neuropharmacology.

[42] García L.M. (2023). Dolor Neuropático periférico en neuropatías por atrapamiento: Fisiopatología y manejo de fisioterapia. J. MOVE Ther. Sci..

