# Supplementary material for: How Does Nerve Mechanical Interface Treatment Impact Pre-Surgical Carpal Tunnel Syndrome Patients? A Randomized Controlled Trial
Source: J Pers Med. 2024 Jul 29;14(8):801. doi: 10.3390/jpm14080801 (PMC11355142; doi:10.3390/jpm14080801)
Supplement: Supplementary file 1 [file jpm-14-00801-s001.zip › jpm-3103479-supplementary.pdf]

SUPLEMENTARY MATERIAL

Table S1. CONSORT statement

Section/Topic	Item No	Checklist item	Reported on page No
**Title and abstract**			
	1a	Identification as a randomised trial in the title	1
	1b	Structured summary of trial design, methods, results, and conclusions (for specific guidance see CONSORT for abstracts)	1
**Introduction**			
Background and objectives	2a	Scientific background and explanation of rationale	1-2
	2b	Specific objectives or hypotheses	2
**Methods**			
Trial design	3a	Description of trial design (such as parallel, factorial) including allocation ratio	2
	3b	Important changes to methods after trial commencement (such as eligibility criteria), with reasons	2
Participants	4a	Eligibility criteria for participants	2-3
	4b	Settings and locations where the data were collected	2-3
Interventions	5	The interventions for each group with sufficient details to allow replication, including how and when they were actually administered	3
Outcomes	6a	Completely defined pre-specified primary and secondary outcome measures, including how and when they were assessed	3-4
	6b	Any changes to trial outcomes after the trial commenced, with reasons	2-3
Sample size	7a	How sample size was determined	5
	7b	When applicable, explanation of any interim analyses and stopping guidelines	-
Randomisation:			
Sequence generation	8a	Method used to generate the random allocation sequence	2-3
	8b	Type of randomisation; details of any restriction (such as blocking and block size)	2-3
Allocation concealmen	9	Mechanism used to implement the random allocation sequence (such as sequentially numbered containers), describing any steps taken to conceal the sequence until interventions were assigned	2-3

t mechanism			
Implementation	10	Who generated the random allocation sequence, who enrolled participants, and who assigned participants to interventions	2-3
Blinding	11a	If done, who was blinded after assignment to interventions (for example, participants, care providers, those assessing outcomes) and how	2-3
	11b	If relevant, description of the similarity of interventions	-
Statistical methods	12a	Statistical methods used to compare groups for primary and secondary outcomes	4-5
	12b	Methods for additional analyses, such as subgroup analyses and adjusted analyses	4-5
**Results**			
Participant flow (a diagram is strongly recommended)	13a	For each group, the numbers of participants who were randomly assigned, received intended treatment, and were analysed for the primary outcome	5 (Figure 1)
	13b	For each group, losses and exclusions after randomisation, together with reasons	5 (Figure 1)
Recruitment	14a	Dates defining the periods of recruitment and follow-up	5 (Figure 1)
	14b	Why the trial ended or was stopped	5 (Figure 1)
Baseline data	15	A table showing baseline demographic and clinical characteristics for each group	Table 1 & S3
Numbers analysed	16	For each group, number of participants (denominator) included in each analysis and whether the analysis was by original assigned groups	Table 2 & S4; Figure 2, 3 & 4
Outcomes and estimation	17a	For each primary and secondary outcome, results for each group, and the estimated effect size and its precision (such as 95% confidence interval)	Table 2 & S4; Figure 2, 3 & 4
	17b	For binary outcomes, presentation of both absolute and relative effect sizes is recommended	Table 2 & S4; Figure 2, 3 & 4
Ancillary analyses	18	Results of any other analyses performed, including subgroup analyses and adjusted analyses, distinguishing pre-specified from exploratory	Table S4 Figure 3
Harms	19	All important harms or unintended effects in each group (for specific guidance see CONSORT for harms)	-
**Discussion**			

Limitations	20	Trial limitations, addressing sources of potential bias, imprecision, and, if relevant, multiplicity of analyses	10
Generalisability	21	Generalisability (external validity, applicability) of the trial findings	10
Interpretation	22	Interpretation consistent with results, balancing benefits and harms, and considering other relevant evidence	9-10
**Other information**			
Registration	23	Registration number and name of trial registry	2
Protocol	24	Where the full trial protocol can be accessed, if available	2
Funding	25	Sources of funding and other support (such as supply of drugs), role of funders	11

.

Figure S1. Physiotherapy intervention

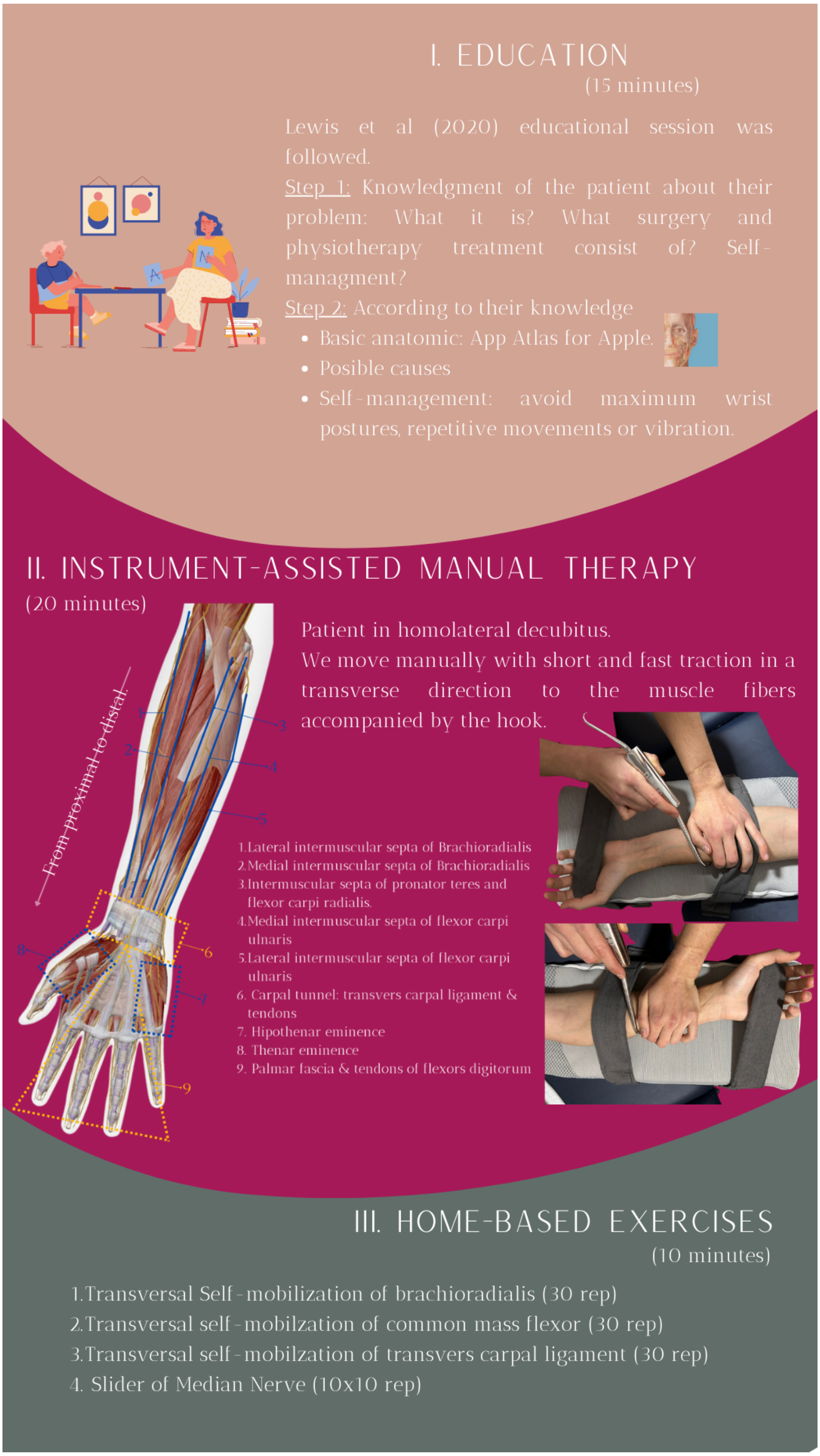

Table S2. Sociodemographic outcomes

Variable	Categories
Sex	Men & Female
Age	Number of Years
Body Mass Index	Kg.m^2
Time with symptoms	Number of years
Severity	Mild, Moderate & Severe
Bilaterality	Yes & No
Ethiology	None, Work, Age, Comorbidities & Other
Comorbidities	Yes & No
Number of comorbidities	None, One & Two or More
Physical Activity	Follow OMS recommendation or not
Actual professio**n**	Active & Inactive
Work status	Employed, Off Work, Unemployed & Retired
Alcohol consumption	Daily, Weekly, Monthly & No
Tabacco consumption	Yes & No

Table S3. Baseline primary and secondary outcomes

	**Intervention Group (n=20)**	**Control Group (n=22)**
Boston Carpal Tunnel Questionnaire		
· *Symptoms Severity Scale (1-5)*	3.09±.84	3.19±.72
· *Functional Severity Scale (1-5)*	2.06±.79	2.54±.60
Paint Intensity (VAS: 0-100 mm)	42.6±25.9	43.7±23.8
Sensitivity of the hand (1.65-6.65)	3.31±.93	3.39±.48
Hand grip strength (Kg)	23.2±10.6	18.5±11.0
ULNT1 (º)	96.6±12.6	90.2±1.1
Tampa Scale for Kinesiophobia (11-44)	26.9±4.6	28.6±8.1

Table S4. Between-group comparisons in primary and secondary outcomes including significant sociodemographic characteristics

		T1 – After Treatment				T2 – 3 Months				T3 – 6 Months			
		Mean±SD	F	p-value	η²	Mean±SD	F	p-value	η²	Mean±SD	F	p-value	η²
		Alcohol Consumption: Daily											
Boston Carpal Tunnel Questionnaire *Symptoms Severity Scale* (1-5)	CG (n=4)	2.98±.61	5.61	**.024†**	0.15	2.72±.51	2.27	.141	0.06	3.05±.56	2.22	.146	0.06
	IG (n=3)	2.21±.56				2.09±.48				2.31±.50			
		Alcohol Consumption: Weekly											
	CG (n=5)	3.13±.26	1.82	.187	0.05	2.55±.69	1.45	.237	0.04	2.65±1.03	1.80	.189	0.05
	IG (n=6)	2.62±.41				2.02±.53				2.03±.68			
		Alcohol Consumption: Monthly											
	CG (n=3)	3.03±.14	5.61	**.024†**	0.15	3.42±.41	5.05	**.032†**	0.13	3.09±.64	4.80	**.036†**	0.13
	IG (n=4)	2.34±1.13				2.50±1.11				2.02±.75			
		Alcohol Consumption: None											
	CG (n=10)	3.37±.80	10.29	**.003**	0.24	3.65±.65	6.22	**.018†**	0.16	3.37±.67	3.38	.075	0.09
	IG (n=7)	2.45±.81				2.78±.88				2.66±.76			
		Actual Profession: Active											
Pain intensity (0-100)	CG (n=)	56.4±22.2	36.90	**<.001†**	0.50	55.2±27.0	12.94	**.001†**	0.26	58.0±21.9	17.10	**<.001†**	0.32
	IG (n=)	18.8±11.4				23.1±18.1				20.4±19.8			
		Actual Profession: Inactive											
	CG (n=)	40.0±22.2	12.20	**.001†**	0.25	52.7±22.4	1.01	.321	0.03	35.8±40.3	4.02	.052	0.10
	IG (n=)	9.9±10.4				38.3±30.3				14.0±15.1			

ULNT1: Upper limb tension test 1. Data are shown as mean±standar deviation. *Comparison between group by repeated-measured general lineal model (p<0.05).
